# Descriptive epidemiology of a cholera outbreak in Kaduna State, Northwest Nigeria, 2014

**DOI:** 10.11604/pamj.2017.27.172.11925

**Published:** 2017-07-04

**Authors:** Ibrahim Baffa Sule, Mohammed Yahaya, Abubakar Ahmed Aisha, Ahmed Datti Zainab, Bajoga Ummulkhulthum, Patrick Nguku

**Affiliations:** 1 Nigeria Field Epidemiology and Laboratory Training Program, Zaria, Nigeria; 2 Aminu Kano Teaching Hospital, Kano, Nigeria; 3 Usmanu Danfodio University, Sokoto, Nigeria; 4 Ahmadu Bello University, Zaria, Nigeria

**Keywords:** Cholera, disease outbreak, epidemics, Nigeria, vibrio cholerae

## Abstract

**Introduction:**

Cholera is an acute gastrointestinal infection caused by Vibrio cholerae, which may lead to severe dehydration and death if not treated. This analysis is aimed at highlighting the magnitude, pattern and trend of cholera outbreak that occurred in Kaduna State in 2014.

**Methods:**

We obtained the 2014 cholera line-list from the Kaduna State Disease Surveillance and Notification officer (DSNO). We described the outbreaks in time, place and person using Epi-info 7 and Health Mapper.

**Results:**

A total of 1468 case-patients and 54 deaths were recorded, giving a case fatality rate (CFR) of 3.68%. Female case-patients were 809(55.08%). The median age for case-patients was 15 years, with an age range of 0.04-90 years. Age specific case fatality rate (ASCFR) is highest among the > 60 years. Seven (30%) out of the 23 local government areas (LGAs) in Kaduna State were affected by the cholera outbreak in 2014. Igabi LGA has the highest attack rate (150.46 per 100,000 population) while Chikun LGA has the lowest attack rate (12.22 per 100,000 population). Chikun LGA records the highest CFR (17.54%). Cholera infection spread across LGAs sharing the same borders. The outbreak started from the first epidemic week of 2014 and lasted over 33 weeks.

**Conclusion:**

Our analysis revealed a protracted cholera outbreak that gradually increases in magnitude throughout the first half of 2014 and spread within contiguous LGAs. We recommended the strengthening of the state's diseases surveillance system towards timely detection and early response to disease outbreaks in the future.

## Introduction

Cholera is an acute gastrointestinal infection caused by Vibrio cholerae, which are curved aerobic bacilli, which are motile, possessing a polar flagellum [1]. The primary symptoms of cholera are profuse diarrhea and vomiting, after an incubation period of about 2 hours to 5 days [2]. Severe cholera, without treatment, kills about 50% of infected patients [2, 3]. A rapid dipstick test is available to determine the presence of V. cholerae, especially in epidemic situations. Treatment is usually started without or before laboratory confirmation. Stool and swab samples collected in the acute stage of the disease, before antibiotics have been administered, are the most useful specimens for laboratory diagnosis [3–5]. Although cholera may be life-threatening, prevention of the disease is normally straight forward if proper sanitation practices are followed [6, 7]. The World Health Organization recommends focusing on prevention, preparedness and response to combat the spread of cholera. They also stress the importance of an effective surveillance system for early detection of potential outbreaks [6]. Cholera has continued to be a global threat to public health and a key indicator of lack of social development [8]. Though used to be a common phenomenon worldwide, it is now largely confined to developing and underdeveloped countries, mainly in the tropical and sub-tropical regions [9]. Cholera is endemic in Africa, parts of Asia, the Middle East, South and Central America [6, 10]. Factors that may lead to outbreaks in endemic areas include, wars, civil unrests, flooding, earthquakes, landslide, poor sanitation and improper refuse dumping, open defecation and slumps [7, 11].

Worldwide, about 1.4-4.3 million cases and 28,000-142,000 deaths per year are due to cholera infection [6, 7]. In 2013, 47 countries reported a total of 129,064 cases of cholera, including 2120 deaths, giving a Case-Fatality Rate (CFR) of 1.63% [7, 10]. These represent a decrease of 47% in the number of cases reported compared to 2012. Cases were reported from all regions of the world, including 22 countries in Africa, 14 in Asia, two in Europe, eight in America, and one from Oceania. Out of the 26 countries that reported deaths from cholera in the same year, 17 were in Africa accounting for 1366 deaths (CFR 2.43%) or 65% of global total [6, 7]. In West Africa, when compared to 2012, the number of cases reported in 2013 showed an important decrease of 80% (from 50,663 to 9765). Only Nigeria and Togo reported more cases than in 2012 [12, 13]. In Nigeria, cholera infection is endemic and outbreaks are common [14]. An upsurge of cholera cases was reported in September, 2013 by the Federal Ministry of Health and continued throughout December, 2013. A total of 6600 cholera cases, including 229 deaths (CFR 3.47%) was reported from 94 Local Government Areas (LGAs) in 20 states (Kaduna state inclusive) [15]. Kaduna State has been in the forefront of most recent cholera outbreaks in Nigeria. In August 1, 2015, an outbreak of suspected cholera cases was reported in Zaria LGA. With more than 40 cases and more than 10 deaths recorded [7, 10]. This study aimed to describe the magnitude, pattern and trends of the cholera outbreak that occurred in Kaduna State in 2014 and to give recommendations to relevant authorities for future prevention of its reoccurrence.

## Methods

**Study setting**: Kaduna State lies in the North-West zone of Nigeria. It is home to more than 59 ethnicities with diverse social, cultural and religious inclinations. It has a total human population of 6.067 million [16]. It consists of 23 Local government areas and divided into three senatorial districts (North, Central and South). The population growth rate was estimated at 3%. The projected population for 2014 was estimated at 7.685 million [16]. Kaduna State has a total of 1580 health facilities scattered across its 23 LGAs. Routine cholera reporting is done through the Integrated Disease Surveillance and Response (IDSR) platform. Cholera outbreaks are submitted as line-list from the LGAs Disease Surveillance and Notification Officers (DSNOs) to the state's DSNO.

**Study design**: The study was a descriptive secondary data analysis. Data was sourced and obtained from the Kaduna State Disease Surveillance and Notification Officer (DSNO) at the Primary Health Care Board and consist of line-list of all reported cholera cases in Kaduna State from January to December, 2014 covering all the 23 LGAs of Kaduna State.

**Data management**: Data was keenly observed and systematically cleaned. The variables of interest collected include, age, sex, date of onset of symptoms, LGAs, wards, outcome and laboratory results. Specific checks were made on coding and categorization of variables, these include harmonizing date formats, using same unit for age and correction of spelling mistakes. Line-list with incomplete data were identified and excluded from the analysis. We calculate frequencies, proportions, attack rates and age specific attack rates and analyzed data in time, place and person using Microsoft Excel 2016, Epi-info 7.2 and health mapper.

**Cholera surveillance in Kaduna Stateb**: Cholera surveillance in Kaduna State is through the IDSR platform. Information flow from the health facilities, through the ward focal persons to the LGA's DSNOs and then to the State's DSNOs. Feedback goes through the opposite direction.

**IDSR cholera case definitions suspected case**: In an area where there is a cholera epidemic, cholera should be suspected in all patients with acute watery diarrheoa.

**Confirmed case**: Any suspected case confirmed by laboratory isolation of vibrio cholera 01 and 0139 from stool sample taken from a patient with acute diarrheoa.

**Probable case**: A clinically compatible case that is epidemiologically linked to a confirmed case.

**Laboratory investigations**: Stool samples were usually tested using a rapid diagnostic testing kit (RDT) for vibrio cholera during a suspected cholera outbreak.

**Ethical issues**: Approval was obtained from the Kaduna State primary health chare board, before data was collected. Ethical clearance was also obtained from Kaduna State Ministry of Health ethical committee.

## Results

A total of 1468 case-patients and 54 deaths (CFR 3.7%) were recorded during the 2014 cholera outbreak in Kaduna State. There were 809(55.1%) Female case-patients and 659(44.9%) male case-patients. The median age for case-patients was 15years, with an age range of 0.04-90.0 years. The majority 865(58.9%) of case-patients were within 1-20 years age group, followed by 21-40 years age group 360(24.5%). This pattern was seen in both sexes. There were more deaths in males 1088(74.1%) than in females 380(25.9%). Age Specific Case Fatality Rate (ASCFR) is highest in the elderly (> 60 years) with ASCFR of 20%, followed by the 41-60 years age group (13.3%). This pattern is also seen in both sexes (Table 1). Only 22 (1.4%) stool samples were recorded to have been test using the cholera RDT kit and 2 (9.1%) of these samples were recorded to have tested positive for cholera. Cholera cases were recorded as onset of symptoms from the first epidemiologic week of 2014. The cholera outbreak spans over a period of 33 epidemiologic weeks. The outbreak occurred through the first half of the year (from January to August). The epidemic pattern shows a propagated outbreak, with the epidemic curve showing two distinct peaks. The first peak was on the 10th epidemiologic week (147 cases and 18 deaths). These cases were reported from five (5) LGAs which are Chikum (20 cases/6 deaths), Igabi (3 cases/2 deaths), Kaduna North (87 cases, no deaths), Soba (14 cases /3 deaths) and Zaria (22 cases/4 deaths).

**Table 1:** Distribution of cholera cases and deaths by age group across local governments areas of Kaduna State, 2014

Age Groups	Male		Female		Total	
	Cases	%	Cases	%	Cases	%
**<1**	59	8.95	54	6.67	113	7.70
**1 - 20**	383	58.12	482	59.58	865	58.92
**21 - 40**	146	22.15	214	26.45	360	24.52
**41 - 60**	58	8.80	47	5.81	105	7.15
**>60**	13	1.97	12	1.48	25	1.70
**Total**	**659 (44.9)**		**809 (55.1)**		**1468**	
**Age Groups**	**Male**		**Female**		**Total**	
	**Deaths**	**ASCFR (%)**	**Deaths**	**ASCFR (%)**	**Deaths**	**ASCFR (%)**
**<1**	2	3.39	1	1.85	3	2.65
**1 - 20**	15	3.92	7	1.45	22	2.54
**21 - 40**	8	5.48	2	0.93	10	2.78
**41 - 60**	11	18.97	3	6.38	14	13.33
**>60**	4	30.77	1	8.33	5	20.00

**Figure 1:**
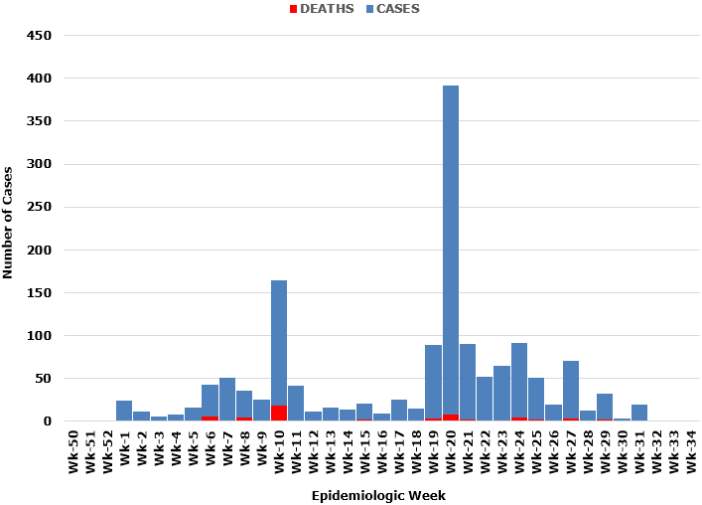
Epidemic curve of the cholera cases and deaths across local government areas of Kaduna State, 2014

**Figure 2:**
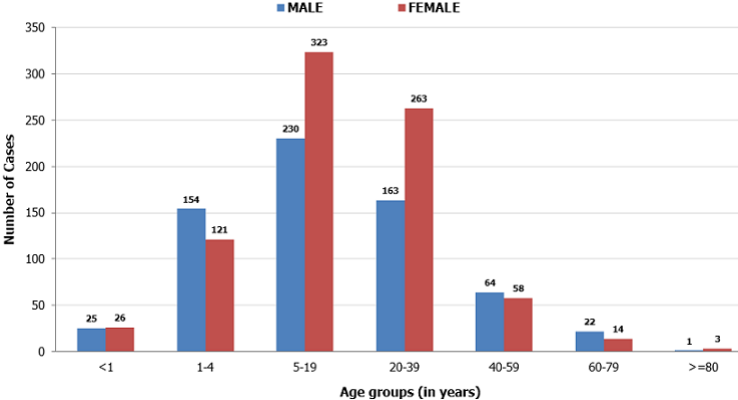
Distribution of cholera cases by age and sex across local government areas of Kaduna State, 2014.

The second peak was at the epidemiologic week 20, with 383 cases and 1 death. These cases were reported from Igabi LGA (298 cases/ 1 death) and Soba LGA (85 cases/ no death). Subsequently, there was a sharp and then a gradual decline in the number of cases, with intermittent rise in number of cases at epidemiologic week 24 (88 cases), week 27 (41 cases) and week 29 (30 cases), the last cases were reported in epidemiologic week 33 (Figure 1). Seven out of the 23 LGAs of Kaduna state were affected during the 2014 cholera outbreak in Kaduna State. The seven LGAs affected were, Chikum, Giwa, Igabi, Kaduna North, Kaduna South, Soba and Zaria. These LGAs had a combined projected population estimate for 2014 of 3,225,777 which is 41.98% of the total estimated population of Kaduna State. The first cases were documented from Kaduna South and Igabi LGAs. Igabi local government reported the highest number of cholera cases (820) within the year 2014 and also has the highest attack rate (150.46 per 100,000 population). Igabi LGA recorded the lowest Case-Fatality Rate (CFR: 1.22%). Conversely, Chikum LGA had the lowest reported cases (57 cases) and lowest attack rate (12.22 per 100,000 population), but had the highest case-fatality rate (17.54%). The highest number of deaths were recorded at Soba LGA (18 deaths), while the lowest number of deaths were recorded at Kaduna South LGA (2 deaths) (Table 2). The temporal spread of the 2014 cholera outbreak shows that no affected LGA was geographically isolated. All affected LGAs were contiguous with one another (shared same boarder). That is, newly affected local governments were situated next to LGAs with previously reported cholera cases. Geographical clustering appears to be in the central and northern senatorial districts of the state (Figure 2).

**Table 2:** Cholera attack rates and case-fatality rates stratified by local governments, Kaduna 2014

LGAs	Cases (%)	Deaths (%)	Projected population 2014	Attack Rate /100,000 pop	CRF (%)
**Chikun**	57 (3.9)	10 (18.5)	466,488	12.22	17.54
**Giwa**	82 (5.6)	5 (9.3)	362,837	22.60	6.10
**Igabi**	820 (55.9)	10 (18.5)	545,001	150.46	1.22
**Kaduna North**	122 (8.2)	5 (9.3)	453,116	26.92	4.10
**Kaduna South**	76 (5.2)	2 (3.7)	509,736	14.91	2.63
**Soba**	244 (16.6)	18(33.3)	371,506	65.68	7.38
**Zaria**	67 (4.6)	4 (7.4)	517,093	12.96	5.97
**Total**	**1468**	**54**	**3,225,777**	**45.51**	**3.68**

## Discussion

This study found that a cholera outbreak in Kaduna State had protracted for seven months of 2014. The outbreak had a CFR above the WHO recommended threshold (1%). Studies have shown fatalities from cholera outbreaks in Nigeria to be reducing, from 7.14% in 2001 to 4.24% in 2010 [17, 18]. This outbreak had a CFR (3.7%) which seems to follow the same trend of reduction. This can be attributed to increase in public awareness and a relative improvement in hygienic practices and environmental sanitary activities. The World Health Organization (WHO) has defined a case of cholera during an epidemic or outbreak as "a patient aged 5 years or more who develops acute watery diarrhoea, with or without vomiting" [8]. But, the IDSR for cholera had the less than 5years age-group in its reporting forms, which has made the IDSR not in alignment with WHO standards. However, the 2014 cholera outbreak in Kaduna State affected patients mostly below the age of 40 years, while fatalities were proportionately more in the elderly population. This may reflect neglect of affected elderly patients, as this is the age-group that rely on relatives and family members for care. More females were affected than males in this outbreak, and there was a documented cholera outbreak at a girl's secondary school (GGSS Kawo) in Kaduna North LGA. The outbreak occurred between 2 to 11 March, 2014 with high number of cases recorded but no recorded death. These confers with earlier studies which showed that more females get infected because in most settings they (the females) take care of the sick at home, hence are more liable to contract the disease [12, 19]. Fatalities was though seen more in males (about trice more) than in the females. The IDSR record showed only about 1.4% of all patients had stool samples tested using the cholera RDT. This record is however dismally low and need to be improved upon. The cholera outbreak in Kaduna State lingered for a prolonged period, spreading across LGAs sharing the same borders. This showed delayed effective response that may have delayed curtailment of the outbreak. Also, the very high attack rates recorded in some LGAs may be due to poor infection control measures at the health facilities. Poor case management at health facilities may be responsible for the high CFR recorded at Chikun LGA.

## Conclusion

Kaduna state experienced a protracted cholera outbreak, especially throughout the first half of 2014. The spread of these outbreak was through contiguous LGAs. We recommended the strengthening of the state's surveillance system and training of health workers for timely detection and effective response to such outbreak. Review and update of IDSR cholera case forms to align with WHO standards. Training of Wards focal persons and LGAs DSNOs on proper data entry and documentation so as to improve on the quality of IDSR data. The provision of drugs and other commodities at health facilities effective case management and public enlightenment campaigns across the state on personal hygiene and proper sanitation.

### What is known about this topic

- Cholera is endemic in most of the sub-saharan Africa sub-region;
- Recurrent outbreak of cholera still occurs in Africa.

### What this study adds

- A protracted cholera outbreak occurred in Kaduna State, affecting one-third of its LGAs and a considerable number of its populace, with a high case fatality rate.

## Competing interests

The authors declare no competing interest.
